# l-2-Nitrimino-1,3-diazepane-4-carboxylic acid monohydrate

**DOI:** 10.1107/S1600536808015146

**Published:** 2008-06-07

**Authors:** Harutyun A. Karapetyan

**Affiliations:** a Molecular Structure Research Center, National Academy of Sciences RA, Azatutyan Ave. 26, 375014 Yerevan, Republic of Armenia

## Abstract

The title compound, C_6_H_10_N_4_O_4_·H_2_O, crystallizes with two independent formula units in the asymmetric unit, their geometric parameters being quite similar. The conformations of the 1,3-diazepane rings are also similar and close to a twist-boat. All ten O- and N-bound H atoms are involved in hydrogen bonds, two of which are intra- and eight inter­molecular linking crystallographically independent mol­ecules, into a three-dimensional hydrogen-bonded network.

## Related literature

For the crystal structures of some analogues of the title compound, see: Apreyan *et al.* (2008*a* ▶, 2008*b* ▶); Karapetyan *et al.* (2007 ▶); Petrosyan *et al.* (2005 ▶); Karapetyan (2008 ▶). For related literature, see: Paul *et al.* (1961 ▶); Apreyan & Petrosyan (2008 ▶).
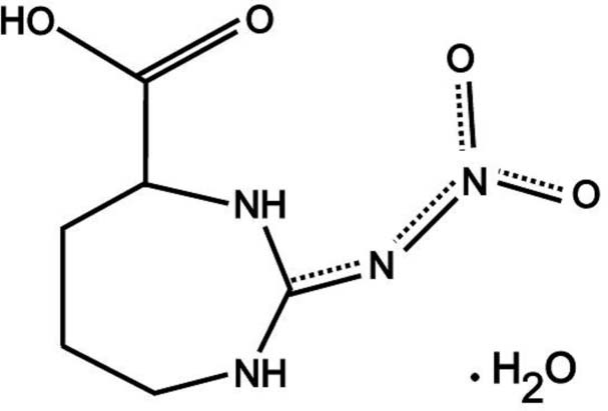


## Experimental

### 

#### Crystal data

C_6_H_10_N_4_O_4_·H_2_O
*M* *_r_* = 220.20
Orthorhombic, 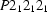

*a* = 9.0115 (18) Å
*b* = 14.729 (3) Å
*c* = 15.257 (3) Å
*V* = 2025.0 (7) Å^3^
*Z* = 8
Mo *K*α radiation
μ = 0.13 mm^−1^
*T* = 293 (2) K
0.22 × 0.17 × 0.12 mm

#### Data collection

Enraf–Nonius CAD-4 diffractometer
Absorption correction: none
6714 measured reflections
2512 independent reflections
1583 reflections with *I* > 2σ(*I*)
*R*_int_ = 0.040
3 standard reflections every 400 reflections intensity decay: none

#### Refinement

*R*[*F* ^2^ > 2σ(*F* ^2^)] = 0.045
*wR*(*F* ^2^) = 0.132
*S* = 1.02
2512 reflections
286 parameters
6 restraints
H atoms treated by a mixture of independent and constrained refinement
Δρ_max_ = 0.44 e Å^−3^
Δρ_min_ = −0.27 e Å^−3^

### 

Data collection: *DATCOL* in *CAD-4 Manual* (Enraf–Nonius, 1988 ▶); cell refinement: *LS* in *CAD-4 Manual* (Enraf–Nonius, 1988 ▶); data reduction: *HELENA* (Spek, 1997 ▶); program(s) used to solve structure: *SHELXS97* (Sheldrick, 2008 ▶); program(s) used to refine structure: *SHELXL97* (Sheldrick, 2008 ▶); molecular graphics: *SHELXTL* (Sheldrick, 2008 ▶); software used to prepare material for publication: *SHELXTL*.

## Supplementary Material

Crystal structure: contains datablocks global, I. DOI: 10.1107/S1600536808015146/bg2187sup1.cif

Structure factors: contains datablocks I. DOI: 10.1107/S1600536808015146/bg2187Isup2.hkl

Additional supplementary materials:  crystallographic information; 3D view; checkCIF report

## Figures and Tables

**Table 1:** Hydrogen-bond geometry (Å, °)

*D*—H⋯*A*	*D*—H	H⋯*A*	*D*⋯*A*	*D*—H⋯*A*
O1—H1⋯N3^i^	0.82	1.90	2.716 (4)	173
N1—H3⋯O3	0.86	2.02	2.586 (4)	123
N2—H10⋯O2^ii^	0.86	2.05	2.889 (4)	163
O5—H11⋯O9^iii^	0.82	1.69	2.510 (5)	174
N5—H13⋯O7	0.86	2.04	2.584 (5)	121
N6—H20⋯O6^iv^	0.86	2.16	2.937 (5)	150
O9—H21⋯N7	0.83 (4)	2.11 (3)	2.902 (6)	160 (7)
O9—H22⋯O10	0.84 (4)	1.86 (3)	2.662 (7)	159 (7)
O10—H23⋯O7^v^	0.86 (4)	2.04 (4)	2.869 (6)	163 (6)
O10—H24⋯O3	0.86 (4)	2.41 (8)	2.856 (6)	113 (5)

Symmetry codes: (i) 
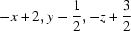
; (ii) 
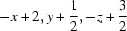
; (iii) 
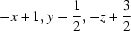
; (iv) 
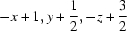
; (v) 
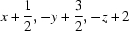
.

**Fig. 1.**
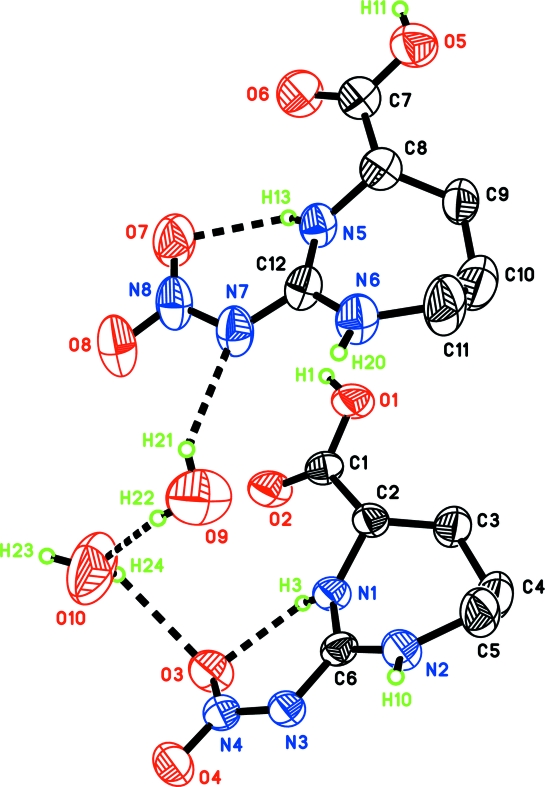
View of the asymmetric unit of (I) showing atomic numbering and displacement ellipsoids at the 50% probability. Only active H atoms are presented for clarity. H-bonds drawn in broken lines.

**Fig. 2.**
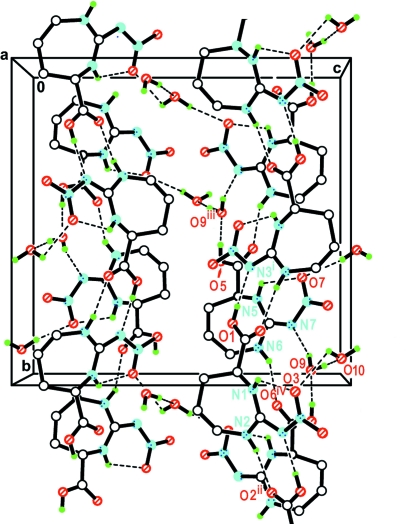
Packing view of the structure ( non-active H atoms not shown). H-bonds drawn in broken lines. Symmetry codes: (i) -x + 2, y - 1/2, -z + 3/2; (ii) -x + 2, y + 1/2, -z + 3/2; (iii) -x + 1, y - 1/2, -z + 3/2; (iv) -x + 1, y + 1/2, -z + 3/2.

## supplementary crystallographic information

### Comment

The L-nitroarginine and its crystaline salts have been investigated as a promising line of non-linear optical materials [Apreyan *et al.*(2008a) and Apreyan *et al.*(2008b)]. The cyclic form of L-nitroarginine was reported for the first time in Paul *et al.*, 1961, where it was suggested to be 2-nitro-4-carboxy-1,3-diazacycloheptane. Recently, on the basis of the crystal structure of the cyclic form of L-nitroarginine [Karapetyan, 2008] it was shown to be L-2-nitrimino-1,3-diazepane-4-carboxylic acid (L-NIDCA).

We present herein a structural study of the L-NIDCA monohydrate, C_6_H_10_N_4_O_4_ × H_2_O (I), which crystallizes with two independent formulas in the asymmetric unit, shown in Fig. 1. The metric parameters of independent L-NIDCA molecules are in agreement with commonly accepted values and their conformations are the same, being close to that of a 7-membered ring twist-boat . All ten active H atoms in the crystal are involved in hydrogen bonding (Table 1), two of them being intra- and eight inter-molecular, linking crystallographically independent units and by way of which a tree-dimensional H bonded network results (Fig. 2).

### Experimental

By the reaction of L-nitroarginine with KOH the potassium salt was obtained. By the interaction of this potassium salt with HBF4 and further separation of the poorly soluble KBF4 salt, single crystals of (I) were obtained by slow evaporation below room temperature. Details of the obtainment of L-NIDCA and L-NIDCA^.^H_2_O, as well as vibrational spectra, thermal properties and SHG will be reported soon separately [Apreyan and Petrosyan, 2008].

### Refinement

The positions of all hydrogen atoms clearly revealed in a difference Fourier map. Foillowing common practice, however, all H atoms except those belonging to water molecules were placed in geometrically calculated positions and included in the refinement in a riding model approximation (O-H: 0.85Å, C-H: 0.97-0.98Å, N-H:0.86Å). The positions of H atoms of both independent water molecules were determined from the difference Fourier maps and refined with restrained O-H: 0.85 (4)Å distances. Displacement parameters were taken as *U*_iso_(H): 1.2*U*_eq_(carrier atom).

In the absense of any significant anomalous effect, Friedel pairs were merged, which explains the rather low parameters/reflections ratio.

### Crystal data

**Table:**

C_6_H_10_N_4_O_4_·H_2_O	*F*(000) = 928
*M**_r_* = 220.20	*D*_x_ = 1.445 Mg m^−^^3^
Orthorhombic, *P*2_1_2_1_2_1_	Mo *K*α radiation, λ = 0.71073 Å
Hall symbol: P 2ac 2ab	Cell parameters from 24 reflections
*a* = 9.0115 (18) Å	θ = 14–16°
*b* = 14.729 (3) Å	µ = 0.13 mm^−^^1^
*c* = 15.257 (3) Å	*T* = 293 K
*V* = 2025.0 (7) Å^3^	Prismatic, yellow
*Z* = 8	0.22 × 0.17 × 0.12 mm

### Data collection

**Table:**

Enraf–Nonius CAD-4 diffractometer	*R*_int_ = 0.040
Radiation source: fine-focus sealed tube	θ_max_ = 27.0°, θ_min_ = 2.6°
graphite	*h* = 0→11
ω/2θ scans	*k* = −17→18
6714 measured reflections	*l* = −19→19
2512 independent reflections	3 standard reflections every 400 reflections
1583 reflections with *I* > 2σ(*I*)	intensity decay: none

### Refinement

**Table:**

Refinement on *F*^2^	Primary atom site location: structure-invariant direct methods
Least-squares matrix: full	Secondary atom site location: difference Fourier map
*R*[*F*^2^ > 2σ(*F*^2^)] = 0.046	Hydrogen site location: inferred from neighbouring sites
*wR*(*F*^2^) = 0.132	H atoms treated by a mixture of independent and constrained refinement
*S* = 1.02	*w* = 1/[σ^2^(*F*_o_^2^) + (0.0704*P*)^2^ + 0.3433*P*] where *P* = (*F*_o_^2^ + 2*F*_c_^2^)/3
2512 reflections	(Δ/σ)_max_ = 0.014
286 parameters	Δρ_max_ = 0.44 e Å^−^^3^
6 restraints	Δρ_min_ = −0.27 e Å^−^^3^

### Special details

**Table:**

Geometry. All e.s.d.'s (except the e.s.d. in the dihedral angle between two l.s. planes) are estimated using the full covariance matrix. The cell e.s.d.'s are taken into account individually in the estimation of e.s.d.'s in distances, angles and torsion angles; correlations between e.s.d.'s in cell parameters are only used when they are defined by crystal symmetry. An approximate (isotropic) treatment of cell e.s.d.'s is used for estimating e.s.d.'s involving l.s. planes.
Refinement. Refinement of *F*^2^ against ALL reflections. The weighted *R*-factor *wR* and goodness of fit *S* are based on *F*^2^, conventional *R*-factors *R* are based on *F*, with *F* set to zero for negative *F*^2^. The threshold expression of *F*^2^ > σ(*F*^2^) is used only for calculating *R*-factors(gt) *etc*. and is not relevant to the choice of reflections for refinement. *R*-factors based on *F*^2^ are statistically about twice as large as those based on *F*, and *R*- factors based on ALL data will be even larger.

### Fractional atomic coordinates and isotropic or equivalent isotropic displacement parameters (Å^2^)

**Table:**

	*x*	*y*	*z*	*U*_iso_*/*U*_eq_	
O1	0.8550 (4)	0.81385 (17)	0.6510 (2)	0.0559 (8)	
H1	0.8648	0.7605	0.6653	0.084*	
O2	1.0592 (4)	0.83261 (16)	0.7324 (2)	0.0556 (8)	
O3	1.2651 (4)	1.00838 (17)	0.83155 (19)	0.0592 (8)	
O4	1.2868 (4)	1.1299 (2)	0.90927 (18)	0.0591 (8)	
N1	1.0627 (4)	1.00945 (18)	0.7113 (2)	0.0421 (7)	
H3	1.1404	0.9789	0.7256	0.051*	
N2	0.9663 (4)	1.1533 (2)	0.6923 (2)	0.0507 (9)	
H10	0.9437	1.2018	0.7207	0.061*	
N3	1.1385 (4)	1.13746 (18)	0.7971 (2)	0.0407 (7)	
N4	1.2311 (4)	1.0889 (2)	0.8470 (2)	0.0431 (8)	
C1	0.9593 (5)	0.8623 (2)	0.6876 (2)	0.0399 (9)	
C2	0.9446 (5)	0.9624 (2)	0.6654 (2)	0.0409 (9)	
H2	0.8492	0.9843	0.6879	0.049*	
C3	0.9495 (6)	0.9789 (3)	0.5664 (2)	0.0561 (11)	
H4	1.0283	0.9428	0.5406	0.067*	
H5	0.8563	0.9601	0.5403	0.067*	
C4	0.9765 (8)	1.0790 (3)	0.5471 (3)	0.0733 (16)	
H6	1.0822	1.0907	0.5512	0.088*	
H7	0.9467	1.0911	0.4872	0.088*	
C5	0.8980 (6)	1.1435 (3)	0.6055 (3)	0.0688 (15)	
H9	0.8951	1.2026	0.5774	0.083*	
H8	0.7964	1.1231	0.6129	0.083*	
C6	1.0582 (5)	1.0969 (2)	0.7326 (2)	0.0382 (8)	
O5	0.2203 (4)	0.6209 (2)	0.6113 (2)	0.0681 (9)	
H11	0.1992	0.5667	0.6112	0.102*	
O6	0.3842 (4)	0.5793 (2)	0.7134 (2)	0.0702 (9)	
O7	0.6264 (5)	0.6721 (2)	0.8634 (2)	0.0816 (12)	
O8	0.7679 (5)	0.7592 (2)	0.9383 (2)	0.0785 (11)	
N5	0.5019 (4)	0.7416 (2)	0.7264 (2)	0.0474 (8)	
H13	0.5467	0.6920	0.7399	0.057*	
N6	0.5183 (5)	0.8983 (2)	0.7299 (2)	0.0581 (10)	
H20	0.5327	0.9434	0.7646	0.070*	
N7	0.6483 (5)	0.8224 (2)	0.8311 (2)	0.0564 (10)	
N8	0.6787 (5)	0.7483 (3)	0.8780 (2)	0.0594 (10)	
C7	0.3282 (5)	0.6353 (3)	0.6665 (3)	0.0518 (10)	
C8	0.3764 (5)	0.7345 (2)	0.6670 (3)	0.0479 (10)	
H12	0.2949	0.7710	0.6908	0.057*	
C9	0.4115 (6)	0.7691 (3)	0.5742 (3)	0.0577 (12)	
H14	0.4718	0.7244	0.5441	0.069*	
H15	0.3195	0.7757	0.5419	0.069*	
C10	0.4921 (8)	0.8586 (4)	0.5744 (3)	0.0860 (17)	
H16	0.5977	0.8462	0.5761	0.103*	
H17	0.4717	0.8888	0.5191	0.103*	
C11	0.4585 (9)	0.9205 (3)	0.6433 (3)	0.0859 (19)	
H18	0.3515	0.9250	0.6482	0.103*	
H19	0.4953	0.9799	0.6267	0.103*	
C12	0.5530 (5)	0.8170 (2)	0.7613 (2)	0.0453 (9)	
O9	0.8626 (5)	0.9582 (3)	0.8857 (4)	0.0980 (13)	
H21	0.799 (5)	0.918 (3)	0.883 (4)	0.118*	
H22	0.927 (6)	0.944 (4)	0.923 (4)	0.118*	
O10	1.1127 (6)	0.9147 (4)	0.9685 (4)	0.134 (2)	
H23	1.132 (9)	0.882 (5)	1.014 (3)	0.161*	
H24	1.170 (9)	0.893 (6)	0.929 (4)	0.161*	

### Atomic displacement parameters (Å^2^)

**Table:**

	*U*^11^	*U*^22^	*U*^33^	*U*^12^	*U*^13^	*U*^23^
O1	0.063 (2)	0.0304 (13)	0.0741 (19)	−0.0079 (14)	−0.0175 (17)	0.0017 (13)
O2	0.0548 (17)	0.0305 (13)	0.081 (2)	0.0015 (13)	−0.0159 (18)	0.0023 (14)
O3	0.0668 (19)	0.0344 (13)	0.0765 (18)	0.0104 (14)	−0.0205 (18)	−0.0031 (13)
O4	0.070 (2)	0.0534 (16)	0.0541 (16)	0.0008 (17)	−0.0194 (16)	−0.0042 (14)
N1	0.0486 (19)	0.0241 (13)	0.0536 (17)	0.0017 (14)	−0.0098 (16)	−0.0009 (13)
N2	0.065 (2)	0.0289 (15)	0.058 (2)	0.0053 (16)	−0.0198 (19)	−0.0017 (14)
N3	0.0478 (19)	0.0276 (14)	0.0467 (16)	0.0026 (15)	−0.0085 (16)	−0.0011 (13)
N4	0.051 (2)	0.0338 (15)	0.0446 (16)	−0.0019 (16)	−0.0056 (17)	0.0014 (13)
C1	0.042 (2)	0.0301 (16)	0.047 (2)	0.0002 (18)	0.0023 (19)	−0.0020 (16)
C2	0.048 (2)	0.0291 (17)	0.0456 (19)	−0.0018 (17)	−0.008 (2)	−0.0025 (15)
C3	0.082 (3)	0.042 (2)	0.045 (2)	−0.008 (2)	−0.006 (2)	−0.0004 (17)
C4	0.117 (5)	0.057 (3)	0.047 (2)	−0.008 (3)	−0.011 (3)	0.008 (2)
C5	0.099 (4)	0.040 (2)	0.068 (3)	0.006 (3)	−0.031 (3)	0.007 (2)
C6	0.046 (2)	0.0289 (16)	0.0402 (18)	−0.0023 (17)	0.0028 (18)	−0.0001 (15)
O5	0.072 (2)	0.0556 (18)	0.077 (2)	−0.0083 (17)	−0.010 (2)	0.0005 (17)
O6	0.086 (2)	0.0405 (15)	0.085 (2)	0.0007 (16)	−0.015 (2)	0.0082 (16)
O7	0.123 (3)	0.0527 (19)	0.069 (2)	−0.007 (2)	−0.022 (2)	0.0136 (16)
O8	0.111 (3)	0.074 (2)	0.0512 (16)	0.024 (2)	−0.021 (2)	−0.0030 (15)
N5	0.060 (2)	0.0358 (15)	0.0461 (18)	0.0087 (16)	−0.0062 (19)	−0.0002 (14)
N6	0.082 (3)	0.0379 (17)	0.055 (2)	0.0094 (18)	−0.006 (2)	−0.0026 (15)
N7	0.081 (3)	0.0446 (18)	0.0435 (17)	0.0112 (19)	−0.010 (2)	−0.0004 (15)
N8	0.082 (3)	0.057 (2)	0.0387 (17)	0.018 (2)	−0.003 (2)	−0.0031 (17)
C7	0.053 (3)	0.047 (2)	0.056 (2)	0.005 (2)	0.000 (2)	−0.003 (2)
C8	0.050 (2)	0.039 (2)	0.055 (2)	0.0087 (19)	0.001 (2)	−0.0025 (18)
C9	0.069 (3)	0.055 (3)	0.049 (2)	0.000 (2)	−0.011 (2)	0.0107 (19)
C10	0.114 (5)	0.084 (4)	0.059 (3)	−0.012 (4)	−0.005 (3)	0.017 (3)
C11	0.140 (6)	0.040 (2)	0.078 (3)	0.008 (3)	−0.035 (4)	0.013 (2)
C12	0.059 (2)	0.0377 (19)	0.0396 (19)	0.009 (2)	0.006 (2)	0.0003 (16)
O9	0.082 (3)	0.059 (2)	0.152 (4)	0.013 (2)	−0.013 (3)	0.009 (2)
O10	0.110 (4)	0.172 (5)	0.120 (4)	0.017 (4)	−0.016 (3)	0.082 (4)

### Geometric parameters (Å, °)

**Table:**

O1—C1	1.306 (5)	O6—C7	1.203 (5)
O1—H1	0.8200	O7—N8	1.238 (5)
O2—C1	1.212 (5)	O8—N8	1.232 (5)
O3—N4	1.247 (4)	N5—C12	1.316 (5)
O4—N4	1.232 (4)	N5—C8	1.453 (5)
N1—C6	1.329 (4)	N5—H13	0.8600
N1—C2	1.450 (5)	N6—C12	1.326 (5)
N1—H3	0.8600	N6—C11	1.463 (6)
N2—C6	1.324 (5)	N6—H20	0.8600
N2—C5	1.467 (5)	N7—N8	1.334 (5)
N2—H10	0.8600	N7—C12	1.371 (6)
N3—N4	1.338 (4)	C7—C8	1.525 (6)
N3—C6	1.359 (5)	C8—C9	1.536 (6)
C1—C2	1.518 (5)	C8—H12	0.9800
C2—C3	1.531 (5)	C9—C10	1.505 (7)
C2—H2	0.9800	C9—H14	0.9700
C3—C4	1.523 (6)	C9—H15	0.9700
C3—H4	0.9700	C10—C11	1.424 (7)
C3—H5	0.9700	C10—H16	0.9700
C4—C5	1.482 (7)	C10—H17	0.9700
C4—H6	0.9700	C11—H18	0.9700
C4—H7	0.9700	C11—H19	0.9700
C5—H9	0.9700	O9—H21	0.83 (4)
C5—H8	0.9700	O9—H22	0.84 (4)
O5—C7	1.304 (5)	O10—H23	0.86 (4)
O5—H11	0.8200	O10—H24	0.86 (4)
			
C1—O1—H1	109.5	C12—N5—C8	125.8 (3)
C6—N1—C2	124.0 (3)	C12—N5—H13	117.1
C6—N1—H3	118.0	C8—N5—H13	117.1
C2—N1—H3	118.0	C12—N6—C11	127.9 (3)
C6—N2—C5	128.3 (3)	C12—N6—H20	116.1
C6—N2—H10	115.9	C11—N6—H20	116.1
C5—N2—H10	115.9	N8—N7—C12	119.9 (3)
N4—N3—C6	120.6 (3)	O8—N8—O7	120.1 (4)
O4—N4—O3	120.8 (3)	O8—N8—N7	115.3 (4)
O4—N4—N3	115.5 (3)	O7—N8—N7	124.6 (4)
O3—N4—N3	123.6 (3)	O6—C7—O5	125.8 (4)
O2—C1—O1	125.4 (3)	O6—C7—C8	122.4 (4)
O2—C1—C2	122.7 (4)	O5—C7—C8	111.8 (4)
O1—C1—C2	111.9 (3)	N5—C8—C7	107.0 (3)
N1—C2—C1	107.0 (3)	N5—C8—C9	113.0 (3)
N1—C2—C3	112.3 (3)	C7—C8—C9	111.9 (3)
C1—C2—C3	111.8 (3)	N5—C8—H12	108.3
N1—C2—H2	108.5	C7—C8—H12	108.3
C1—C2—H2	108.5	C9—C8—H12	108.3
C3—C2—H2	108.5	C10—C9—C8	112.9 (4)
C4—C3—C2	110.4 (3)	C10—C9—H14	109.0
C4—C3—H4	109.6	C8—C9—H14	109.0
C2—C3—H4	109.6	C10—C9—H15	109.0
C4—C3—H5	109.6	C8—C9—H15	109.0
C2—C3—H5	109.6	H14—C9—H15	107.8
H4—C3—H5	108.1	C11—C10—C9	117.3 (5)
C5—C4—C3	115.4 (4)	C11—C10—H16	108.0
C5—C4—H6	108.4	C9—C10—H16	108.0
C3—C4—H6	108.4	C11—C10—H17	108.0
C5—C4—H7	108.4	C9—C10—H17	108.0
C3—C4—H7	108.4	H16—C10—H17	107.2
H6—C4—H7	107.5	C10—C11—N6	116.5 (4)
N2—C5—C4	113.9 (4)	C10—C11—H18	108.2
N2—C5—H9	108.8	N6—C11—H18	108.2
C4—C5—H9	108.8	C10—C11—H19	108.2
N2—C5—H8	108.8	N6—C11—H19	108.2
C4—C5—H8	108.8	H18—C11—H19	107.3
H9—C5—H8	107.7	N5—C12—N6	122.2 (4)
N2—C6—N1	120.9 (4)	N5—C12—N7	125.7 (3)
N2—C6—N3	113.2 (3)	N6—C12—N7	112.1 (3)
N1—C6—N3	125.9 (3)	H21—O9—H22	109 (5)
C7—O5—H11	109.5	H23—O10—H24	104 (5)
			
C6—N3—N4—O4	−172.7 (4)	C12—N7—N8—O8	178.5 (4)
C6—N3—N4—O3	9.7 (6)	C12—N7—N8—O7	0.0 (7)
C6—N1—C2—C1	−156.2 (3)	C12—N5—C8—C7	−162.6 (4)
C6—N1—C2—C3	80.7 (5)	C12—N5—C8—C9	73.8 (5)
O2—C1—C2—N1	−3.2 (5)	O6—C7—C8—N5	4.4 (6)
O1—C1—C2—N1	178.4 (3)	O5—C7—C8—N5	−176.7 (3)
O2—C1—C2—C3	120.1 (4)	O6—C7—C8—C9	128.7 (5)
O1—C1—C2—C3	−58.3 (5)	O5—C7—C8—C9	−52.4 (5)
N1—C2—C3—C4	−44.5 (6)	N5—C8—C9—C10	−45.9 (5)
C1—C2—C3—C4	−164.7 (4)	C7—C8—C9—C10	−166.7 (4)
C2—C3—C4—C5	−39.4 (7)	C8—C9—C10—C11	−32.6 (7)
C6—N2—C5—C4	−20.4 (7)	C9—C10—C11—N6	73.8 (8)
C3—C4—C5—N2	77.0 (6)	C12—N6—C11—C10	−26.1 (9)
C5—N2—C6—N1	−22.8 (7)	C8—N5—C12—N6	−16.8 (7)
C5—N2—C6—N3	159.5 (4)	C8—N5—C12—N7	164.7 (4)
C2—N1—C6—N2	−19.6 (6)	C11—N6—C12—N5	−18.4 (8)
C2—N1—C6—N3	157.9 (4)	C11—N6—C12—N7	160.3 (5)
N4—N3—C6—N2	176.6 (3)	N8—N7—C12—N5	−10.4 (6)
N4—N3—C6—N1	−1.0 (6)	N8—N7—C12—N6	170.9 (4)

### Hydrogen-bond geometry (Å, °)

**Table:**

*D*—H···*A*	*D*—H	H···*A*	*D*···*A*	*D*—H···*A*
O1—H1···N3i	0.82	1.90	2.716 (4)	173.
N1—H3···O3i	0.86	2.02	2.586 (4)	123.
N2—H10···O2i	0.86	2.05	2.889 (4)	163.
O5—H11···O9i	0.82	1.69	2.510 (5)	174.
N5—H13···O7i	0.86	2.04	2.584 (5)	121.
N6—H20···O6i	0.86	2.16	2.937 (5)	150.
O9—H21···N7i	0.83 (4)	2.11 (3)	2.902 (6)	160 (7)
O9—H22···O10i	0.84 (4)	1.86 (3)	2.662 (7)	159 (7)
O10—H23···O7i	0.86 (4)	2.04 (4)	2.869 (6)	163 (6)
O10—H24···O3i	0.86 (4)	2.41 (8)	2.856 (6)	113 (5)

Symmetry codes: i; i; i; i; i; i.

## References

[bb1] Apreyan, R. A., Karapetyan, H. A. & Petrosyan, A. M. (2008*a*). *J. Mol. Struct.* **874**, 187–193.

[bb2] Apreyan, R. A., Karapetyan, H. A. & Petrosyan, A. M. (2008*b*). *J. Mol. Struct.* **875**, 272–281.

[bb3] Apreyan, R. A. & Petrosyan, A. M. (2008). In preparation.

[bb4] Enraf–Nonius (1988). *CAD-4 Manual.* Enraf–Nonius, Delft, The Netherlands.

[bb5] Karapetyan, H. A. (2008). *Acta Cryst.* E**64**, o943. 10.1107/S1600536808011835 PMC2961324 21202424

[bb6] Karapetyan, H. A., Antipin, M. Yu., Sukiasyan, R. P. & Petrosyan, A. M. (2007). *J. Mol. Struct.* **831**, 90–96.

[bb7] Paul, R., Anderson, G. W. & Callahan, F. M. (1961). *J. Org. Chem.* **26**, 3347–3350.

[bb8] Petrosyan, A. M., Sukiasyan, R. P., Karapetyan, H. A., Antipin, M. Yu. & Apreyan, R. A. (2005). *J. Cryst. Growth*, **275**, e1927–e1933.

[bb9] Sheldrick, G. M. (2008). *Acta Cryst.* A**64**, 112–122. 10.1107/S0108767307043930 18156677

[bb10] Spek, A. L. (1997). *HELENA* University of Utrecht, The Netherlands.

